# Observation of Agonistic Behavior in Pacific White Shrimp (*Litopenaeus vannamei*) and Transcriptome Analysis

**DOI:** 10.3390/ani14111691

**Published:** 2024-06-05

**Authors:** Bo Wu, Chenxi Zhao, Xiafei Zheng, Zhilan Peng, Minhai Liu

**Affiliations:** 1 Ninghai Institute of Mariculture Breeding and Seed Industry, Zhejiang Wanli University, Ningbo 315000, China; wubo@zwu.edu.cn (B.W.); 2021900025@zwu.edu.cn (C.Z.); zhengxiafei@zwu.edu.cn (X.Z.); 2 Zhejiang Engineering Research Center for Aquacultural Seeds Industry and Green Cultivation Technologies, College of Biological and Environmental Sciences, Zhejiang Wanli University, Ningbo 315000, China; pengzhilan@zwu.edu.cn

**Keywords:** shrimp behavior, behavior model, differentially expressed genes, eyestalk, brain ganglion

## Abstract

**Simple Summary:**

Agonistic behavior plays a crucial role in managing intraspecific competition among crustaceans. To explore the characteristics and regulatory mechanisms of agonistic behavior in *L. vannamei*, we quantified this behavior using a behavioral observation system and employed RNA-seq methods to characterize differentially expressed genes (DEGs) between aggressive and non-aggressive groups. The results revealed that *L. vannamei* exhibits nine correlated behavior patterns and the fighting process followed a specific process. Energy metabolism and signal transduction pathways may be key factors influencing agonistic behavior in shrimp. Additionally, the eyestalk may play a crucial role in initiating agonistic behavior.

**Abstract:**

Agonistic behavior has been identified as a limiting factor in the development of intensive *L. vannamei* aquaculture. However, the characteristics and molecular mechanisms underlying agonistic behavior in *L. vannamei* remain unclear. In this study, we quantified agonistic behavior through a behavioral observation system and generated a comprehensive database of eyestalk and brain ganglion tissues obtained from both aggressive and nonaggressive *L. vannamei* employing transcriptome analysis. The results showed that there were nine behavior patterns in *L. vannamei* which were correlated, and the fighting followed a specific process. Transcriptome analysis revealed 5083 differentially expressed genes (DEGs) in eyestalk and 1239 DEGs in brain ganglion between aggressive and nonaggressive *L. vannamei*. Moreover, these DEGs were primarily enriched in the pathways related to the energy metabolism process and signal transduction. Specifically, the phototransduction (dme04745) signaling pathway emerges as a potential key pathway for the adjustment of the *L. vannamei* agonistic behavior. The *G protein-coupled receptor kinase 1-like* (*LOC113809193*) was screened out as a significant candidate gene within the phototransduction pathway. Therefore, these findings contribute to an enhanced comprehension of crustacean agonistic behavior and provide a theoretical basis for the selection and breeding of *L. vannamei* varieties suitable for high-density aquaculture environments.

## 1. Introduction

Agonistic behavior represents a principal form of social interaction among crustaceans, serving as a mechanism to navigate intraspecific competition [1]. This behavior is crucial for survival and reproduction, as it facilitates the maintenance of dominance, defense of territories, monopolization of resources and mates, and protection of offspring [2,3,4]. However, these aggressive encounters are energetically costly, leading to significant energy expenditure and potential resource depletion [5]. Additionally, frequent aggressive interactions can cause physical harm or even result in mortality, emphasizing the complex balance between the benefits and costs of agonistic behavior.

The Pacific white shrimp, *L. vannamei*, stands as the most prominent species in global shrimp aquaculture due to its rapid growth rate, strong adaptability, and extensive distribution [6]. It is recognized as one of the foremost cultured shrimp species worldwide. Group living inherently brings about conflict and competition. Notably, cannibalistic and agonistic behaviors are frequently reported in shrimp cultures, posing significant challenges [7,8], in particular as the costs associated with shrimp farming rise and aquaculture technologies advance. Consequently, intensive, high-density aquaculture practices have become popular [9,10], but they also exacerbate issues such as inter-individual aggression and self-mutilation, which cannot be ignored [11]. In conditions of high stocking density, competition for essential survival resources intensifies [12], leading to increased size disparity among the population, decreased survival rates, and diminished farming efficiency [13,14]. Therefore, understanding how to mitigate agonistic behavior in *L. vannamei* within intensive, high-density farming settings is crucial for enhancing aquaculture productivity. A comprehensive understanding of the characteristics and regulatory mechanisms behind agonistic behavior is essential to address this issue effectively.

Traditionally, research on agonistic behavior in shrimp has primarily been conducted under laboratory conditions using methods such as direct visual observation, video recordings with subsequent playback analysis, and video recordings paired with image software analysis in glass aquaria or specially designed translucent tanks [15,16,17,18]. Concurrently, researchers have applied statistical analysis and mathematical modeling to construct animal behavioral models [17,19]. Presently, studies investigating the agonistic behavior of *L. vannamei* primarily focus on environmental factors such as density, feed type, and temperature, in addition to individual characteristics such as sex and body size [20,21,22,23]. However, these investigations tend to only skim the surface regarding the characteristics and patterns of agonistic behavior, offering limited insight into the underlying molecular regulatory mechanisms. While it has been demonstrated that agonistic behavior among *L. vannamei* individuals significantly influences phenotypic traits and is heritable [14], a comprehensive understanding of the molecular underpinnings remains unclear, with few studies addressing related genes. The advent of genomic technologies has led to the successful sequencing of the *L. vannamei* genome, providing high-quality reference maps and facilitating transcriptome analysis [24]. Such analysis allows for the identification of DEGs associated with specific phenotypes or functions and a deeper understanding of their mechanisms and interactions [25]. For example, transcriptomic studies under agonistic conditions in *Fenneropenaeus chinensis* have identified candidate genes associated with agonistic behavior, including *CASK-interacting protein 2* (*Caskin-2*, *CK*) and *Calcineurin* (*CN*) [26].

The objective of this paper is to develop a quantitative model of agonistic behavior in *L. vannamei*, elucidating associated behavior patterns. We employed transcriptomic analysis to examine the transcripts from the eyestalk and brain ganglion tissues of aggressive and nonaggressive *L. vannamei*, aiming to identify the key regulatory pathways and genes associated with agonistic behavior. Furthermore, this study aims to provide a theoretical framework for the development of new shrimp varieties that are tolerant to high-density aquaculture conditions.

## 2. Materials and Methods

### 2.1. Experimental Animal Collection and Maintenance

Healthy *L. vannamei*, with intact limbs and a mean body weight of 6.83 ± 1.73 g, were obtained from the Ninghai Research Centre of the East China Sea Fisheries Research Institute, Chinese Academy of Fisheries Sciences. The shrimp were housed in 1000-L PVC tanks for maintenance. To minimize stress, the shrimp were acclimatized to the experimental conditions for one week before the commencement of the study. The seawater used was natural, subjected to sedimentation and sand filtration processes, with salinity maintained at 22.0 ± 1.0, water temperature at 29.0 ± 1.0 °C, and pH at 7.8 ± 0.2. The shrimp were fed compound feed at 8:00 and 18:00 daily. Residual feed and feces were removed at 6:00 each following day, and half of the seawater was replaced daily to maintain optimal living conditions. 

### 2.2. Laboratory Experiments

#### 2.2.1. Experimental Equipment

**Figure 1:**
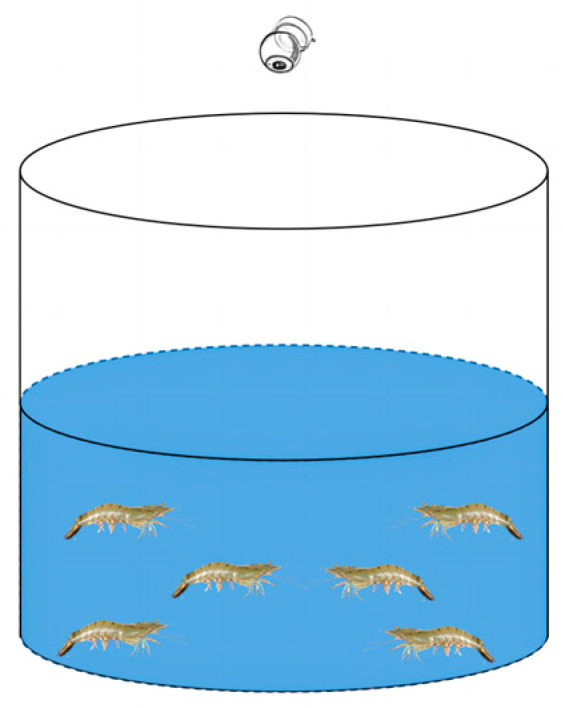
Diagram of the experimental equipment used for studying the agonistic behavior of *L. vannamei*.

For the experimental setup, the video capture system used was model DS-2CD3T46 (Hikvision, Hangzhou, China), which was equipped with 8-million-pixel star-level EXIR infrared lattice cameras. These cameras offer a maximum frame rate of 60 Hz and an image resolution of up to 3840 × 2160 pixels (4K) (Figure 1). The experimental tanks had dimensions of 60 cm in diameter and 100 cm in height. 

#### 2.2.2. Data Acquisition and Statistical Analysis

The video capture system DS-2CD3T46 (Hikvision, China) was used to record the behavioral videos of 150 healthy shrimp. To ensure that the behavior of each shrimp could be clearly observed, every six shrimp were divided into a group and eight hours of behavioral videos were recorded. The recorded videos were carefully reviewed using the Media Recorder software (VSPlayer V7.4.0). Drawing from the methodologies established in our prior experiments, the recorded videos were meticulously reviewed frame by frame. This process enabled the documentation and quantitative analysis of the frequency of agonistic behaviors exhibited by the shrimp in each group. To delineate the sequence structure of the agonistic behavior in *L. vannamei*, we developed a behavioral model diagram using a statistical model, specifically the Markov chain model, integrated with the statistical data collected from the observations.

Formulae: The probability of transitioning to the next behavior can be represented by *p*_ij_ (*p*_ij_ = *p*(E_i_→E_j_) = *p*(X_n+1_|X_n_), 0 ≤ *p* ≤ 1). In this model, we consider that the behavior of the shrimp can occupy one of *N* distinct states, E_i_ (i = 1, 2,..., *N*). We denote the state variable as X_n_ to indicate that t_n_ is in the E_i_ state, and the probability of transition to the E_j_ state at t_n+1_ is *p*_ij_.

### 2.3. RNA-Seq Experiment

#### 2.3.1. Sample Collection

Using both instantaneous and continuous observation methods, three rounds of screening were conducted to categorize shrimp into those exhibiting agonistic behavior (aggressive group) and those displaying no such behavior while maintaining normal activity (non-aggressive group). Over a span of three days, approximately 300 healthy shrimp were used to screen for the aggressive and non-aggressive groups. Three rounds of screening were conducted by three experienced observers for 8 h per day. In each round of screening, shrimp that all actively attacked were in the aggressive group, and shrimp that none attacked were in the non-aggressive group (Table 1).

In the aggressive group, eyestalk (AE) and brain ganglion (AB) were harvested from 60 selected shrimp. Each 20 shrimp was considered one biological replicate, and there were three replicates in total. These tissues were treated with diethylpyrocarbonate (DEPC) water, were subsequently transferred into 1.5 mL sampling tubes, immediately frozen in liquid nitrogen, and then stored at −80 °C. Similarly, in the non-aggressive group, eyestalk (NE) and brain ganglion (NB) from another set of 60 shrimp were processed identically. Each 20 shrimp was considered one biological replicate, and there were three replicates in total. The prepared tissue samples from both groups were packaged in dry ice and dispatched to Beijing Novogene Technology Co., Ltd. (Beijing, China) for the construction of the cDNA library and subsequent sequencing analysis.

#### 2.3.2. RNA Extraction, cDNA Library Construction, and Illumina Sequencing

The eyestalks and brain ganglia of each group of 20 shrimp were mixed into one sample each. There were 3 samples per tissue in each group. Total RNA from each sample was extracted using the TRIzol reagent (Invitrogen, Carlsbad, CA, USA). The quality and purity of the RNA samples were evaluated using two methods: (1) Purity checks were checked by 1% *w*/*v* agarose gel electrophoresis. (2) The concentration of the total RNA was determined using a Nanodrop 2000 spectrophotometer (Thermo Scientific, Wilmington, DE, USA). Subsequently, 12 samples (AE, AB, NE, and NB), each containing 6 mg of total RNA, were packaged in dry ice and sent to Novogene (Novogene Technology Corporation, Beijing, China) for the cDNA library construction and Illumina sequencing. Then, high-throughput RNA sequencing was performed to obtain raw reads, which are publicly accessible in the Sequence Read Archive (SRA-NCBI) under the accession number SRP475566 (BioProject PRJNA1048175).

#### 2.3.3. Quality Control

The fastp software (https://github.com/OpenGene/fastp, accessed on 1 May 2024) was used to remove reads containing undetermined bases, low-quality bases, and adapter contamination, with the process adhering to default parameters to ensure the generation of clean reads. Concurrently, the quality metrics, including Q20, Q30, and GC content, were calculated for these clean reads. All subsequent analyses were conducted using these high-quality, clean reads to ensure the integrity and reliability of the results. Moreover, to analyze the overall transcript level changes between the two groups, principal component analysis (PCA) was performed on the gene expression data from all samples. 

#### 2.3.4. Reads Mapping to the Reference Genome

Reference genome and gene model annotation files were directly retrieved from the appropriate genome database. The clean reads obtained were aligned to the reference genome of *L. vannamei* (scaffold N50605.56 Kb) using Hisat2 version 2.0.5 [24].

#### 2.3.5. DEG Analysis

Read quantification for each gene was performed using featureCounts version 1.5.0-p3. Subsequently, gene expression levels were normalized using the fragments Per kilobase of exon model per million mapped fragments (FPKM) methodology [27]. Differential expression analysis between the two groups was conducted using the DESeq2 R package version 1.20.0 based on the negative binomial generalized linear models. The *p*-value was adjusted to account for multiple hypothesis testing. Genes were identified as DEGs between the samples based on an adjusted *p*-value < 0.05 and |log_2_fold change| >1 [28].

#### 2.3.6. GO and KEGG Enrichment Analysis of DEGs

Gene Ontology (GO) and Kyoto Encyclopedia of Genes and Genomes (KEGG) enrichment analysis of the DEGs were conducted using the cluster Profiler R package. In this analysis, the GO and KEGG annotations of all genes in *L. vannamei* served as the reference gene sets database. The significance level for this enrichment analysis was established with an adjusted *p*-value threshold of < 0.05 and |log_2_fold change| >1.

### 2.4. Quantitative Real-Time PCR (qPCR) Verification

Ten candidate DEGs were selected at random for validation through qPCR. The 6 DEGs of them (*LOC113811845*, *LOC113811105*, *LOC113806160*, *LOC113816327*, *LOC113816237*, and *LOC113806595*) were selected from the eyestalk RNA-seq data. The 4 DEGs of them (*LOC113811118*, *LOC113830189*, *LOC113816208*, and *LOC113804544*) were selected from the brain ganglia RNA-seq data. The same eyestalk and brain ganglia samples as for RNA-seq were selected for total RNA extraction. Total RNA extraction was performed as previously described, and first-strand cDNA synthesis was conducted using 1 μg of total RNA and the Prime Script RT Reagent Kit (TaKaRa Biotech, Mountain View, CA, USA). The resulting cDNA was diluted to 0.1 mg/mL for qPCR analysis, which was carried out using the CFX96 Touch™ Real-Time PCR system (Bio-Rad, Hercules, CA, USA) and SYBR PreMix Ex Taq (TaKaRa, Dalian, China). Gene-specific primers for each of the ten candidate DEGs were designed using the Primer Premier 6.0 software. The *18S* rRNA gene was employed as an internal reference gene. Details of all primer pairs are listed in Table 2. Each sample underwent three independent biological replicates. The relative expression of the genes was calculated employing the 2^−ΔΔCt^ method [29]. The qPCR results were then compared with those from the transcriptome analysis to validate the expression of the selected genes.

## 3. Results

### 3.1. Description of the Agonistic Behavior of L. vannamei

**Figure 2:**
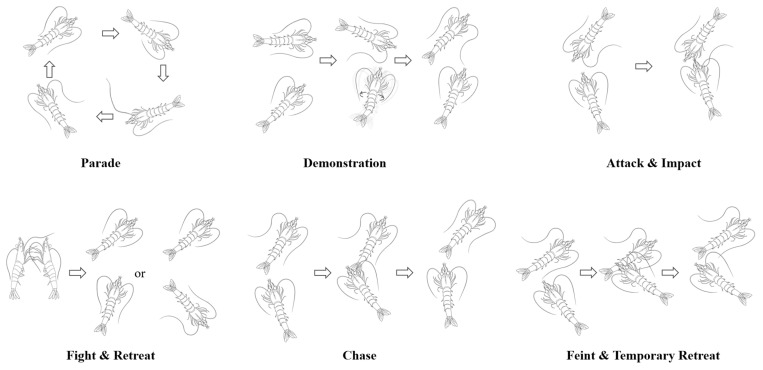
Diagram of the agonistic behavior patterns. Note: (1) Through meticulous and repeated observations of the shrimp fighting videos, the agonistic behavior of the shrimp was categorized into nine behavior patterns. (2) The arrow indicates the order in which the behavior occurs.

Through meticulous and repeated observations of the shrimp fighting videos, the agonistic behavior of the shrimp was categorized into nine behavior patterns: parade, demonstration, attack, fight, chase, impact, feint, temporary retreat, and retreat (Figure 2). These patterns were defined as follows:

1. Parade: A shrimp moves around the bottom of the tank, frequently contacting the tank wall with its flagellum.
2. Demonstration: The shrimp swims back and forth near its opponent while twisting its body or making sudden, quick lunges at the opponent to intimidate and cause the opponent to retreat.
3. Attack: The shrimp approaches its opponent and makes direct physical contact.
4. Fight: Two shrimp confront each other head-on, using their front pereiopods to scratch at the opponent while their pleopods rapidly paddle to advance their bodies until one shrimp retreats or is pushed back.
5. Chase: A shrimp follows its opponent, scratching at the opponent’s body with the first three pairs of pereiopods and sometimes hitting the opponent’s body, continuing until the opponent retreats.
6. Impact: A shrimp quickly moves toward its opponent, striking the opponent from the front or side with its rostrum and occasionally using its antennae to nudge the opponent multiple times.
7. Feint: A shrimp gradually approaches its opponent, tentatively using its pereiopods to scratch at the opponent’s body, which may lead to a retreat or escalate the confrontation.
8. Temporary Retreat: The shrimp contracts its abdomen and jumps backwards, distancing itself temporarily from its opponent in preparation for a subsequent attack.
9. Retreat: The shrimp contracts its abdominal flexors and oblique extensors forcefully while using its uropod to push water forward, propelling its body quickly backward.

### 3.2. Analysis of Agonistic Behavior and Establishment of the Markov Chain Model

**Figure 3:**
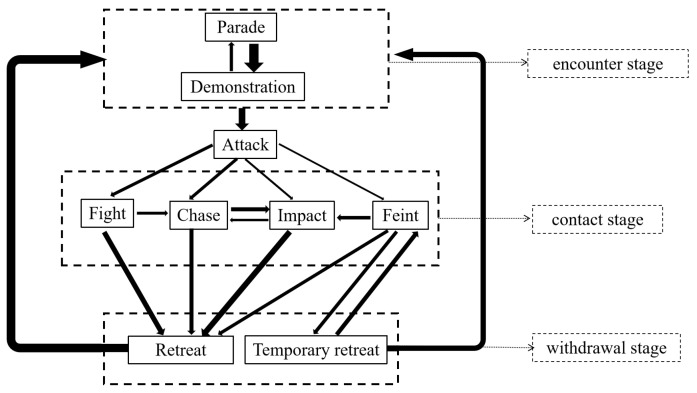
Agonistic behavior model for *L. vannamei*. Note: (1) The Markov chain model was used to establish the relationship between the nine behavior patterns of *L. vannamei* based on Table 3 and Table 4. (2) The arrow points to the next action, and the thickness represents the probability. (3) The dotted boxes represent the three stages according to the purpose of fighting.

Based on extensive video analysis of *L. vannamei*, details regarding the sequence of these behavior patterns were documented. Subsequently, the probabilities of the next behavior pattern were calculated (Table 3). The probability and orientation of the next behavior pattern in the nine behavior patterns were different, as detailed in Table 3. For example, “fight”, “chase”, “impact”, and “feint” may all lead “retreat”, but the probability of occurrence varied from 35.68% to 76.01%. Additionally, these behaviors were categorized into three stages according to the purpose of fighting: “encounter stage” (parade and demonstration), “contact stage” (attack, fight, chase, impact, and feint), and “withdrawal stage” (temporary retreat and retreat), which is useful for analyzing the logic underlying the fighting process of *L. vannamei* (Table 4). Furthermore, the Markov chain model was used to delineate the connections between the various agonistic behaviors of *L. vannamei,* and an agonistic behavioral model diagram was developed based on the data in Table 3 (Figure 3). This diagram was to better understand the mutual orientation between the different agonistic behaviors of *L. vannamei*.

### 3.3. RNA-Seq Data

A total of 12 samples underwent sequencing, yielding 91.36 Gb of clean data. The effective data volume for each sample varied between 6.37 and 9.36 Gb. The base quality, measured by the Q30 value, ranged from 91.73% to 93.68%, while the average GC content was recorded at 47.39%. The Illumina sequencing data were mapped to the reference genome of *L. vannamei*, resulting in 78–90% of the reads from the two tissues aligning specifically to the reference genome. This high rate of specific alignment indicated that the sequencing quality was sufficient to meet the criteria for the subsequent bioinformatics analyses (Supplemental Table S1).

### 3.4. Principal Component Analysis of Sequencing Data

**Figure 4:**
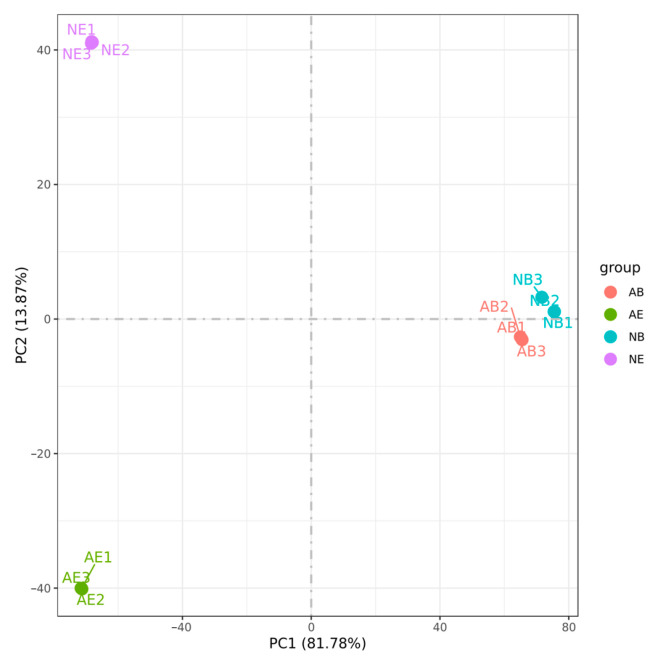
Principal component analysis (PCA) of the aggressive and non-aggressive groups. Note: (1) Different colors represent different comparing groups; (2) brain ganglion (AB, *n* = 3) and eyestalk (AE, *n* = 3) in the aggressive group; brain ganglion (NB, *n* = 3) and eyestalk (NE, *n* = 3) in the non-aggressive group.

The results demonstrated that the samples with three biological replicates consistently clustered together among the 12 samples, indicating high reproducibility of the transcriptome data across different replicates and ensuring the reliability of the experimental data. Furthermore, the transcriptome data from the same tissue types within the samples were consistently grouped together, with the brain ganglion of the two groups showing the closest proximity (Figure 4).

### 3.5. DEG Analysis

**Figure 5:**
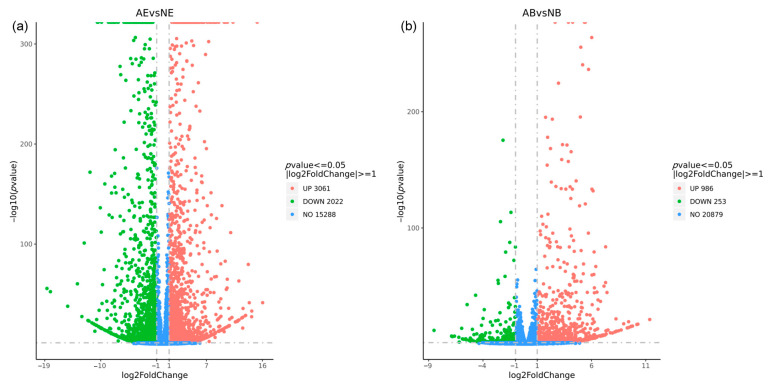
Statistics of differentially expressed genes in aggressive and non-aggressive groups. Note: (**a**): AE vs. NE; (**b**): AB vs. NB; the *x*-axis represents the logarithm value of the difference multiple of gene expression with 2 as the base, the *y*-axis represents the negative logarithm value of the *p*-value with 10 as the base; the significance level for this analysis was established with a *p*-value threshold of < 0.05 and |log_2_fold change| >1; the left (green dot) indicates down-regulated genes; the right (red dot) indicates up-regulated genes; the middle (blue dot) indicates the genes with no significant difference.

The differential expression analysis revealed 5083 DEGs in the eyestalk, with 3061 DEGs being up-regulated and 2022 DEGs down-regulated (Figure 5a). In contrast, the brain ganglion exhibited 1239 DEGs, with 986 up-regulated and 253 down-regulated (Figure 5b). The analysis clearly indicated that the number of DEGs in the eyestalk was higher than in the brain ganglion.

### 3.6. GO Enrichment Analysis of DEGs

**Figure 6:**
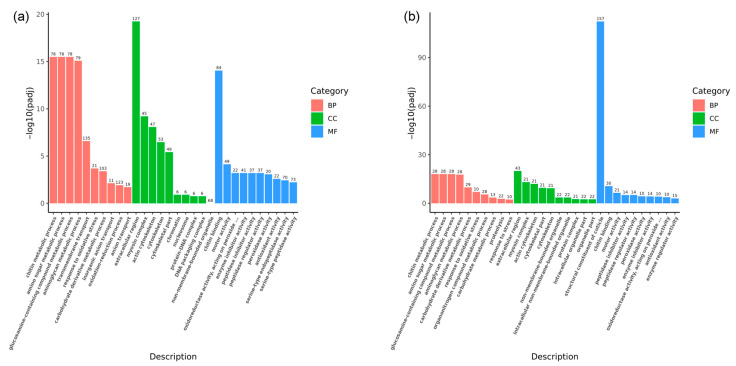
GO annotation for differentially expressed genes in aggressive and non-aggressive groups. Note: (**a**): AE vs. NE; (**b**): AB vs. NB; Gene Ontology (GO) enrichment analysis of the DEGs was conducted using the cluster Profiler R package; most DEGs can be divided into three major categories, including biological process (BP), cellular component (CC), and molecular function (MF) as are shown in different colors; the *x*-axis represents the name of the pathway; the *y*-axis represents the average −log_10_ (adjusted *p*-value); the higher the column, the more significant the pathway; the number on the column represents the number of genes in the pathway.

GO enrichment analyses were conducted to delve deeper into the biological functions of these DEGs during the fighting process. The analyses identified 687 GO terms associated with the eyestalk (Figure 6a). Within the biological process (BP) category, the most significant terms were chitin metabolic process (78 DEGs, GO:0006030), amino sugar metabolic process (78 DEGs, GO:0006040), glucosamine-containing compound metabolic process (78 DEGs, GO:1901071), aminoglycan metabolic process (79 DEGs, GO:0006022), and transmembrane transport (135 DEGs, GO:0055085), with transmembrane transport showing the highest DEG count. In the cellular component (CC) category, the extracellular region (127 DEGs, GO:0005576), myosin complex (45 DEGs, GO:0016459), and actin cytoskeleton (47 DEGs, GO:0015629) were most represented. Chitin binding (84 DEGs, GO:0008061) was the most notable term within molecular function (MF), followed by motor activity (49 DEGs, GO:0003774) and oxidoreductase activity (22 DEGs, GO:0016684). For the brain ganglion, 226 GO terms were identified (Figure 6b). The three most significant terms within biological process were chitin metabolic process (28 DEGs, GO:0006030), amino sugar metabolic process (28 DEGs, GO:0006040), and glucosamine-containing compound metabolic process (28 DEGs, GO:1901071). In the cellular component, the extracellular region (43 DEGs, GO:0005576) emerged as the most significant, followed by the myosin complex and actin cytoskeleton (21 DEGs each, GO:0016459 and GO:0015629). Within MF, the structural constituent of the cuticle (157 DEGs, GO:0042302), chitin-binding (30 DEGs GO:0008061), and motor activity (21 DEGs, GO:0003774) were the most prominent terms.

### 3.7. KEGG Enrichment Analysis of DEGs

**Figure 7:**
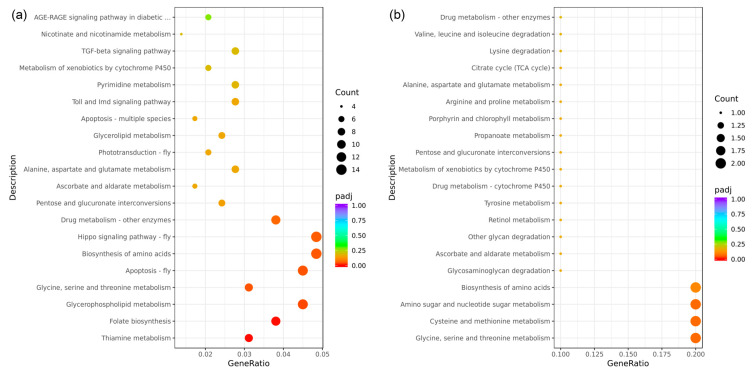
Bubble diagram of the KEGG enrichment analysis of the differentially expressed genes in aggressive and non-aggressive groups. Note: (**a**): AE vs. NE; (**b**): AB vs. NB; the Kyoto Encyclopedia of Genes and Genomes (KEGG) enrichment analysis of the DEGs was conducted using the cluster Profiler R package; the size of the adjusted *p*-value is represented by the color of the dots; the smaller the adjusted *p*-value, the closer the color is to red; the number of differentially expressed genes contained in each pathway is represented by the size of the dots; the *x*-axis represents the gene ratio; the *y*-axis represents the name of the pathway.

In this study, all DEGs identified in the eyestalk were mapped onto reference pathways in the KEGG database to elucidate the biological pathways in which these genes may be involved. The analysis selected the top 20 enriched pathways for further evaluation, as depicted in the KEGG enrichment bubble map (Figure 7). A total of 289 DEGs from the eyestalk were associated with 99 distinct KEGG pathways (Figure 7a). Among the top 20 enriched pathways, only seven were significantly enriched. These significantly enriched pathways included thiamine metabolism (9 DEGs, dme00730), folate biosynthesis (11 DEGs, dme00790), glycerophospholipid metabolism (13 DEGs, dme00564), glycine, serine, and threonine metabolism (9 DEGs, dme00260), apoptosis (13 DEGs, dme04214), and the biosynthesis of amino acids (14 DEGs, pathway code: dme01230). Moreover, while not reaching statistical significance, some signaling pathways exhibited notable enrichment, including the Hippo signaling pathway (14 DEGs, dme04391), phototransduction (6 DEGs, dme04745), Toll and Imd signaling pathway (8 DEGs, dme04624), and the TGF-beta signaling pathway (8 DEGs, dme04350). In contrast, the analysis of DEGs from the brain ganglion revealed that these were mapped to 24 different KEGG pathways, involving 27 DEGs (Figure 7b). However, none of these pathways were significantly enriched, with most relating to energy metabolism, such as amino sugar and nucleotide sugar metabolism (2 DEGs, dme00520), glycosaminoglycan degradation (1 DEG, dme00531), and other glycan degradation (1 DEG, dme00511).

### 3.8. qPCR Verification

**Figure 8:**
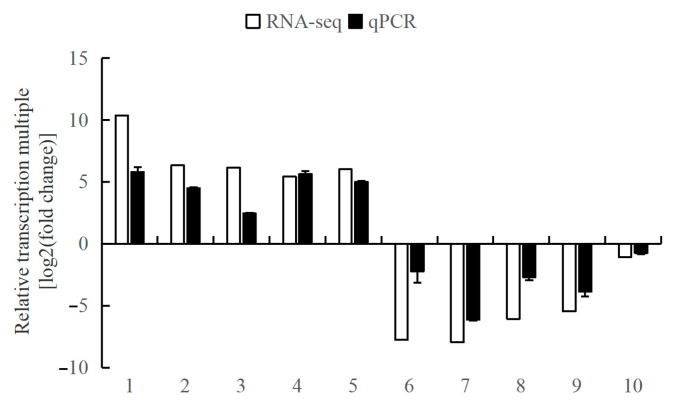
The comparison of the relative transcriptional multiple of qPCR and transcriptome in aggressive and non-aggressive groups. Note: (1) The *x*-axis indicates the gene names; the *y*-axis represents the value of log2(fold change) of the aggressive group compared with the non-aggressive group. (2) *18S* is the internal reference gene; 1. *LOC113811845*, 2. *LOC113811105*, 3. *LOC113806160*, 4. *LOC113811118*, 5. *LOC113830189*, 6. *LOC113816327*, 7. *LOC113816237*, 8. *LOC113806595*, 9. *LOC113816208*, and 10. *LOC113804544*.

In this study, ten DEGs (eyetalks: 6 DEGs; brain ganglia: 4 DEGs), including five up-regulated and five down-regulated, were randomly selected from the two tissues’ RNA-seq data for the validation of the Illumina sequencing results by qPCR analysis. The relative transcriptional multiple of these ten genes, as determined by qPCR, were consistent with those obtained from RNA-seq, as depicted in Figure 8. These results confirm the reliability of the RNA-seq data and validate its use for subsequent analyses.

## 4. Discussion

As shrimp aquaculture intensifies, there has been an observed increase in the frequency of agonistic behavior among individuals in confined spaces [30]. It has been documented that such behavior not only significantly impacts the phenotypic traits of individual shrimp but is also heritable, affecting their progeny [14]. Additionally, *L. vannamei* has gained prominence as a model organism in crustacean biology, particularly since it was among the first crustaceans to have its genome sequenced [24]. Therefore, investigations into the agonistic behavior of *L. vannamei* are of widespread importance. In this research, we utilized behavioral and transcriptomic methods to explore the regulatory mechanisms underlying agonistic behavior in *L. vannamei*, examining both phenotypic traits and molecular genetics. The goal is to provide both a theoretical and molecular foundation for mitigating agonistic behavior in shrimp.

### 4.1. Characteristics of Agonistic Behavior in L. vannamei

Decapod crustaceans exhibit similarities in their behavior patterns. Building on the authors’ previous research on the agonistic behavior of *Portunus trituberculatus* (*P. trituberculatus*) [17], this study identifies and characterizes nine specific actions in the agonistic behavior of *L. vannamei*. Unlike *P. trituberculatus*, known for its sand-diving behavior and daytime activity, captive shrimps remain active continuously [31,32]. The physical distinctions between shrimps and crabs lead to unique manifestations of their agonistic behaviors. For instance, crabs, such as *P. trituberculatus*, use their prominent chelipeds as weapons to strike, grasp, and push opponents during conflicts. In contrast, shrimps, lacking prominent chelipeds, utilize their sharp rostrums and antennae for hitting opponents during altercations. Observations from shrimp litters indicate that they also engage in confrontations using their frontals to hit each other [33], in addition to employing their pereiopods for holding or scratching opponents. Unlike crabs, which retreat using their pereiopods, shrimps execute strong contractions of abdominal flexors to propel their bodies sideways and use their sharply flexed abdomen to stab opponents with their flagellum. Agonistic behaviors in crustaceans typically comprise a sequence of actions, each indicating a specific stage of interaction [27]. In this study, the agonistic behavior model of *L. vannamei* was established by analyzing these nine actions through a Markov chain approach (Figure 2), facilitating the quantitative analysis of their conflict behaviors. The confrontational process in *L. vannamei* can be broadly segmented into three phases: 1. The “encounter stage”, primarily involving non-contact behaviors. Shrimps, being highly active, frequently engage underwater, significantly increasing the likelihood of encounters. They often swim around their opponents to assess the opponent’s resource-holding potential (RHP) and determine whether to escalate the conflict. 2. The “contact stage”, primarily involving contact behaviors. Shrimps of similar sizes often engage in intense confrontations [34] characterized by aggressive actions such as fighting, impacting, and chasing, typically resulting in a clear distinction between winners and losers after several intense rounds. 3. The “withdrawal stage”, where the loser retreats while the winner tends to remain stationary or moves slowly. This phase marks the conclusion of the conflict, leading both parties back to the “encounter stage”.

Similar to *P. trituberculatus*, the agonistic behavior of *L. vannamei* is multifaceted and varies greatly. However, a notable distinction lies in the duration of conflict; *L. vannamei* typically resolve their disputes in a single round, whereas *P. trituberculatus* may engage in multiple rounds. In *L. vannamei*, the outcome of a conflict remains unpredictable until the “withdrawal stage” is reached, contrasting with *P. trituberculatus*, where winners exhibit distinct behaviors, such as climbing onto the loser’s cephalothorax or engaging in persistent chasing, compelling the loser to retreat repeatedly. Furthermore, the agonistic actions of *L. vannamei* exhibit a structured sequence, showing significant interrelationships rather than occurring randomly. For example, a significant correlation exists between the parade and demonstration actions during the “encounter stage”, and escalation to fighting typically occurs only when the RHP of the contestants are closely matched. In the field of scientific ethology, the establishment of behavioral standards and models is crucial, as they enable the acquisition of objective and reliable experimental data [35,36]. Hence, devising a clear and rational model for the agonistic behavior of *L. vannamei* is crucial for advancing crustacean behavioral studies. However, the lack of standardized methodologies in crustacean behavioral studies is evident. Researchers use varied experimental methods and equipment, leading to potential biases influenced by subjective human judgment [37]. The development of new crustacean behavioral recording devices in the future, capable of quantifying behavioral traits numerically or hierarchically, would greatly assist in establishing uniform standards, thereby facilitating the advancement of crustacean behavioral research.

### 4.2. RNA-Seq of L. vannamei Analysis

In this study, DEGs were identified in the eyestalk and brain of *L. vannamei* from both the experimental and control groups using transcriptomic techniques. The findings revealed that the eyestalk exhibited a significantly higher number of DEGs (5083) compared to the brain ganglion (1239). This suggests a notable association between the eyestalk and the manifestation of agonistic behavior. These results align with the findings from studies on *Eriocheir sinensis*, where the removal of eyestalks significantly altered the crabs’ agonistic behavior, particularly causing an increase in such behavior [38]. This comparison provides a valuable point of reference for our further exploration into the role of eyestalk in modulating agonistic behavior in *L. vannamei*. Additionally, the higher number of up-regulated DEGs in both tissues (eyestalk: 3061; brain ganglion: 986) relative to down-regulated DEGs (eyestalk: 2022; brain ganglion: 253) suggests that the up-regulation of gene expression may be a dominant mechanism involved in the regulation of agonistic behavior in these tissues. Of course, down-regulated genes also account for a portion of DEGs, and the specific regulatory mechanisms of agonistic behavior need to be verified in further studies.

In this study, bioinformatics analyses, including GO terms and KEGG pathways, were conducted on DEGs derived from the eyestalk and brain ganglion of *L. vannamei*. The analyses revealed that these DEGs are predominantly involved in the energy metabolism process and signal transduction pathways, suggesting a close relationship between the agonistic behavior of shrimp and their levels of energy metabolism and signal transduction rates. This concept aligns with the theory that combat among individuals essentially constitutes an energy competition, with the outcomes linked to metabolic levels [39,40]. Consistent with the findings from previous studies on crustaceans, individuals exhibiting higher levels of energy metabolism tended to display more aggressive behaviors [41], a pattern also observed in other species such as the brown trout, *Salmo trutta*, where higher energy metabolism correlates with increased aggression and elevated social status [42]. Beyond energy metabolism, a significant portion of the top 20 enriched pathways were associated with signal transduction mechanisms. Behavioral alterations are frequently connected to signal transduction processes [43]. Particularly, in this study, DEGs related to phototransduction (6 DEGs, dme04745) were found to be significantly up-regulated. Phototransduction, the conversion of light photons into electrical signals, is a fundamental process influencing various behaviors in crustaceans, including phototaxis, feeding, and agonistic activities [44,45,46,47,48], suggesting a potential linkage between phototransduction and agonistic behavior. Furthermore, phototransduction involves G-protein-mediated signaling processes [49], and the significant up-regulation of the *G protein-coupled receptor kinase 1-like* (*LOC113809193*), identified in this pathway, implies its involvement in regulating agonistic behaviors. The eyestalk ganglions, located within the eyestalk of crustaceans, are instrumental in both phototransduction and energy metabolism [50]. The data presented herein further substantiate the crucial role of the eyestalk in modulating agonistic behavior. Given the complexity of agonistic behavior regulation in *L. vannamei* and the fact that several of the RNA-Seq identified genes are novel or of unknown function, there is a pressing need to further investigate the specific genetic mechanisms controlling this behavior in *L. vannamei*.

## 5. Conclusions

In this research, we delineated and categorized nine specific actions related to the agonistic behavior of *L. vannamei* into three sequential stages: “encounter”, “contact”, and “retreat”. These behavior patterns were found to be interrelated, with confrontations following a distinct sequence. The eyestalk may play a crucial role in initiating agonistic behavior. DEGs in the eyestalk primarily influence this behavior through the modulation of energy metabolism and signal transduction pathways, with the phototransduction pathway emerging as a potential key factor. Our findings provide valuable insights that could contribute to the theoretical foundation for mitigating agonistic behavior, thereby facilitating the advancement of high-density aquaculture practices for shrimp. However, the study of shrimp behavior lacks a unified standard, and the behavioral regulation within shrimp represents a complex biological process. As such, comprehensively deciphering the characteristics and regulatory mechanisms underlying agonistic behavior in shrimp remains a significant, long-term challenge.

## Figures and Tables

**Table 1:** Screening of the aggressive and non-aggressive groups.

	Day 1 (Round 1)	Day 2 (Round 2)	Day 3 (Round 3)
Aggressive group	+	+	+
Non-aggressive group	−	−	−

Note: Three rounds of screening by instantaneous and continuous observation methods; “+” indicates agonistic behavior; “−” indicates no agonistic behavior.

**Table 2:** Genes and specific primers used for validation of RNA-seq data by real-time PCR.

Primer Name	Sequence (5′–3′)
*LOC113811845*-F	AAATTAGGAGACGCCATGAATC
*LOC113811845*-R	TCAACGCCCAAGCCAGAG
*LOC113811105*-F	GGTCTTCATCCTCCTTGGTC
*LOC113811105*-R	TTGGCTTCTCCTGACTCGTA
*LOC113806160*-F	AAGGTTATTACGCCGACACTG
*LOC113806160*-R	GGTACTGCTGGTTGAAGATGG
*LOC113816327*-F	TACGCAAGGGAGCCACTAAC
*LOC113816327*-R	ACGGCAACTAATGGAAGCAA
*LOC113816237*-F	AAAACCCAACCCTCCCTCTC
*LOC113816237*-R	GCAACATCGTCGCCTAATCC
*LOC113806595*-F	CGATTCCAACCCGTGTCCTC
*LOC113806595*-R	TGCTCCTTCACCCTTCACAC
*LOC113811118*-F	CTCGCAACAACGACAACACT
*LOC113811118*-R	AATGGAACGCAGGAGTCAAA
*LOC113830189*-F	CATCCGCCTCCAGTTCGTG
*LOC113830189*-R	TGGTCGTCGCTTCTTAGGG
*LOC113816208*-F	AGGGAATGGTGGCTCTGTCG
*LOC113816208*-R	CAATGGGTCCTGCTGGGATA
*LOC113804544*-F	TTCCTCTGCCCGTTCCTAAA
*LOC113804544*-R	CTGTGAGCCTCCACCGTAAT
*18S*-F	TATACGCTAGTGGAGCTGGAA
*18S*-R	GGGGAGGTAGTGACGAAAAAT

**Table 3:** Probability of the next pattern after different agonistic behaviors (%).

Behavior	Parade	Demonstration	Attack	Fight	Chase	Impact	Feint	Temporary Retreat	Retreat
Parade		100							
Demonstration	30.08		69.92						
Attack				33.09	33.84	18.04	15.03		
Fight					32.82				67.18
Chase						50.60			49.40
Impact					23.99				76.01
Feint						40.12		24.20	35.68
Temporary retreat		45.47					54.53		
Retreat	52.21	47.79							

**Table 4:** The occurrence probability of each behavior in different fighting stages (%).

Behavior	Encounter Stage	Contact Stage	Withdrawal Stage
Parade	100		
Demonstration	30.08	69.92	
Attack		100	
Fight		32.82	67.18
Chase		50.60	49.40
Impact		23.99	76.01
Feint		40.12	59.88
Temporary retreat	45.47	54.53	
Retreat	100		

## Data Availability

This study was reviewed by the Institutional Animal Care and Use Committee (IACUC) of Zhejiang Wanli University, China. All procedures in this study were in accordance with the ethical standards of the IACUC. The data presented in this study are available in the article. Further information is available upon request from the corresponding author.

## Supplementary Materials

The following supporting information can be downloaded at: https://www.mdpi.com/article/10.3390/ani14111691/s1, Table S1: Quality of transcriptome sequencing samples of *L. vannamei* with fighting.
