# Supplementary material for: Observation of Agonistic Behavior in Pacific White Shrimp (Litopenaeus vannamei) and Transcriptome Analysis
Source: Animals (Basel). 2024 Jun 5;14(11):1691. doi: 10.3390/ani14111691 (PMC11171402; doi:10.3390/ani14111691)
Supplement: Supplementary file 1 [file animals-14-01691-s001.zip › animals-3025360-supplementary.pdf]

Table S1. Quality of transcriptome sequencing samples of *L. vannamei* with fighting.

Sample	raw reads	Clean reads	clean bases	Q20 percentage	Q30 percentage	GC content	Total mapped reads (%)	Uniquely mapped reads (%)
AE1	51725042	50807898	7.62G	96.92	92.39	50.44	39738538(78.21%)	31976077(62.94%)
AE2	50262322	49405998	7.41G	96.98	92.49	50.58	38738689(78.41%)	31098303(62.94%)
AE3	43225084	42450022	6.37G	96.78	92.1	50.4	33074720(77.91%)	26527936(62.49%)
AB1	50668188	49739952	7.46G	96.89	92.14	45.18	43891816(88.24%)	39351797(79.12%)
AB2	48905446	48183048	7.23G	96.85	92.04	44.99	42497976(88.2%)	38082523(79.04%)
AB3	51406264	50637790	7.6G	97.03	92.43	44.94	44824293(88.52%)	40232604(79.45%)
NE1	45564530	44800664	6.72G	96.69	91.91	49.62	35258257(78.7%)	30491630(68.06%)
NE2	59977654	58956536	8.84G	96.7	91.89	49.49	46503118(78.88%)	40173302(68.14%)
NE3	63530672	62387450	9.36G	96.9	92.35	49.62	49221637(78.9%)	42427016(68.01%)
NB1	51846802	51047686	7.66G	96.68	91.73	44.6	44899168(87.96%)	40323534(78.99%)
NB2	55354384	54436142	8.17G	97.64	93.68	44.65	48768833(89.59%)	43809886(80.48%)
NB3	46930904	46131566	6.92G	97.54	93.4	44.13	41419155(89.78%)	37211040(80.66%)
